# Access to Cancer Surgery in a Universal Health Care System During the COVID-19 Pandemic

**DOI:** 10.1001/jamanetworkopen.2021.1104

**Published:** 2021-03-11

**Authors:** Antoine Eskander, Qing Li, Julie Hallet, Natalie Coburn, Timothy P. Hanna, Jonathan Irish, Rinku Sutradhar

**Affiliations:** 1 ICES, Toronto, Ontario, Canada; 2 Institute of Health Policy, Management, and Evaluation, University of Toronto, Ontario, Canada; 3 Department of Otolaryngology–Head and Neck Surgery, University of Toronto, Ontario, Canada; 4 Department of Surgery, University of Toronto, Ontario, Canada; 5 Department of Radiation Oncology, Queen’s University, Kingston, Ontario, Canada; 6 Department of Otolaryngology–Head and Neck Surgery, Sunnybrook Health Sciences Centre, Toronto, Ontario

## Abstract

This cohort study quantifies the cancer surgical backlog and determines whether there were differences in sociodemographic and hospital characteristics among patients undergoing cancer surgery in the pre– and peri–coronavirus disease 2019 (COVID-19) periods.

## Introduction

For many cancers, surgery is central to diagnosis and treatment and is the only curative modality. Treatment delay can result in a missed opportunity for cure and can worsen outcomes.^1,2,3^ Postponing cancer surgery may cost more lives than can be saved by diverting surgical resources and services to managing coronavirus disease 2019 (COVID-19) infection.^1,2^ Delays in surgical care and a backlog of new cancer diagnoses will place unprecedented pressures on health care systems, particularly those with a limited ability to increase throughput.^4^ Data are lacking on the effect of pandemic deferral policies on cancer surgery case volumes and whether specific subgroups have been disproportionately affected. These data are required to inform surgical policies during future waves of the COVID-19 pandemic, ensuring optimal outcomes and equitable care.^4^ Although sociodemographic factors (such as belonging to a minority racial or ethnic group, having low income, and having a nonrural residence) have been associated with increased COVID-19 infection rates and less access to treatment,^5,6^ little is known about whether these factors were also associated with access to cancer surgery during the pandemic. Therefore, we sought to quantify the cancer surgical backlog and determine whether there were differences in sociodemographic and hospital characteristics among patients undergoing cancer surgery in the pre– and peri–COVID-19 periods.

## Methods

This was a population-based retrospective cohort study in Ontario, Canada, that was approved by the Sunnybrook Health Sciences Centre research ethics board. This study followed the Strengthening the Reporting of Observational Studies in Epidemiology (STROBE) reporting guideline. Informed consent was waived because this was a population-based retrospective study. The weekly volume of a comprehensive and well-defined list of cancer-directed hospital-based surgical procedures was determined using Canadian Institute for Health Information procedure codes between January 7, 2018, and June 27, 2020. Only institutions that provided complete data during this period were included in the study.

Segmented regression models were constructed to quantify (1) the surgical volume trend pre–COVID-19 (January 7, 2018, to March 14, 2020; preperiod slope), (2) the immediate decrease in surgical volume at the start of the pandemic (March 15, 2020; change in intercept), and (3) the surgical volume trend during the peri–COVID-19 period (periperiod slope, March 15 to June 27, 2020). In comparing patient characteristics from the pre–COVID-19 period with the peri–COVID-19 period, a standardized difference of greater than 0.1 was used to indicate a meaningful difference between groups. Statistical analysis was performed using SAS Enterprise Guide 7.15 (SAS Institute).

## Results

We included 543 751 patients (mean [SD] age, 56.9 [16.9] years; 332 156 [61.1%] women) from 112 of 120 hospitals (93.3%) eligible for analysis. There was an immediate 60% decrease in the mean surgical volume on March 15, 2020, compared with the mean surgical volume in the pre–COVID-19 period. This decrease was followed by a 6% increase in mean surgical volume each subsequent week. Surgical volumes did not return to pre–COVID-19 numbers by June 27, 2020 (Figure), resulting in 35 671 fewer completed surgical procedures in the peri–COVID-19 period than the pre–COVID-19 period.

**Figure.**
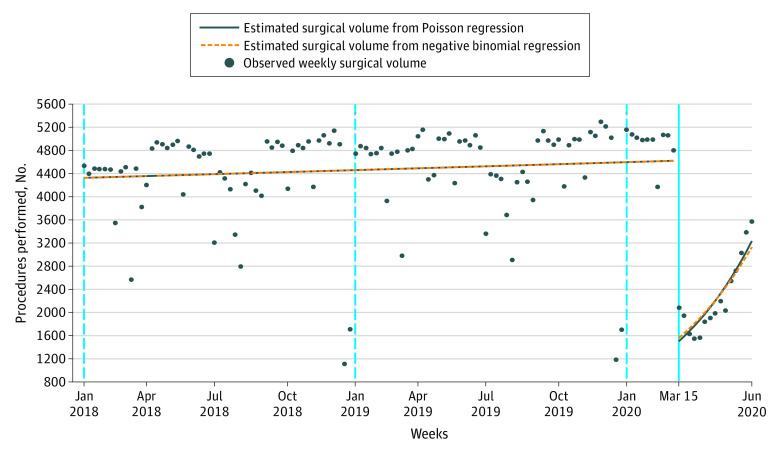
Weekly Cancer-Directed Surgical Procedures, January 2018 to June 2020

There were few sociodemographic differences between the patients who received surgery in the pre– or peri–COVID-19 period (Table). Measures, such as material deprivation, rurality, immigration, and region, did not differ between patients treated in the pre– or peri–COVID-19 period. However, compared with the pre-COVID-19 period, cancer surgery in the peri–COVID-19 period was more often considered urgent (78 263 [15.4%] vs 9365 [27.6%]; standardized difference, 0.30) and performed more frequently at teaching hospitals (360 807 [29.2%] vs 11 628 [34.2%]; standardized difference, 0.11) and in inpatient settings (233 522 [45.8] vs 20 700 [60.9]; standardized difference, 0.31).

**Table.** Distributions of Sociodemographic and Hospital Characteristics for Surgical Procedures Performed During the Pre– and Peri–COVID-19 Periods

Variable	Patients by COVID-19 period, No. (%)		Standardized difference^a^
	Pre (n = 509 786)	Peri (n = 33 965)	
Age, mean (SD), y	56.78 (16.90)	58.63 (17.04)	0.11
Women	312 552 (61.3)	19 604 (57.7)	0.07
Rural score^b^			
0-9, less rural	332 226 (65.2)	21 659 (63.8)	0.03
10-30	93 834 (18.4)	6429 (18.9)	0.01
31-50	55 143 (10.8)	3912 (11.5)	0.02
51-70	15 767 (3.1)	1050 (3.1)	0
≥71, more rural	7252 (1.4)	514 (1.5)	0.01
Immigrant	74 573 (14.6)	4233 (12.5)	0.06
Elixhauser grouping^c^			
0	72 035 (14.1)	4681 (13.8)	0.01
1	45 554 (8.9)	3655 (10.8)	0.06
2	29 242 (5.7)	2358 (6.9)	0.05
≥3	39 585 (7.8)	3335 (9.8)	0.07
No hospitalization	323 370 (63.4)	19 936 (58.7)	0.10
Material deprivation quintile^d^			
1, least deprived	110 549 (21.7)	7375 (21.7)	0
2	106 727 (20.9)	6923 (20.4)	0.01
3	98 176 (19.3)	6468 (19.0)	0.01
4	95 078 (18.7)	6440 (19.0)	0.01
5, most deprived	95 586 (18.8)	6495 (19.1)	0.01
Region			
Toronto	32 819 (6.4)	2142 (6.3)	0.01
Central	145 795 (28.6)	8769 (25.8)	0.06
East	133 403 (26.2)	9135 (26.9)	0.02
North	39 807 (7.8)	2870 (8.4)	0.02
West	157 962 (31.0)	11 049 (32.5)	0.03
Inpatient surgery^e^	233 522 (45.8)	20 700 (60.9)	0.31
Nonteaching hospital status	360 807 (70.8)	22 337 (65.8)	0.11
Urgent^f^	78 263 (15.4)	9365 (27.6)	0.30^g^

Abbreviation: COVID-19, coronavirus disease 2019.

^a^ Standardized difference of greater than 0.1 was used to indicate a clinically and statistically significant imbalance in the distributions of the characteristics.

^b^ Rural Score was based on the Rurality Index for Ontario version 2008. This measure took into account (1) community population and population density, (2) travel time to nearest basic referral center, and (3) travel time to nearest advanced referral center and was updated based on the most recent census data.

^c^ The Elixhauser comorbidity grouping was calculated using a 5-year look-back window in administrative data for any hospitalization. This is a well-validated approach to assess comorbidities using administrative data. Although no hospitalization is grouped with 0 in prior studies, these categories were separated here to provide additional information on comorbidity variation for the reader.

^d^ Material deprivation is a composite measure of socioeconomic status that takes into account the proportion of a population that is without a high school diploma, families who are lone parent families, receiving government transfer payments, unemployed, considered low income, and living in dwellings that are in need of major repair.

^e^ This variable is a measure of procedures that were performed on an inpatient basis as opposed to same-day surgery (same-day discharge or discharge from a short-stay unit after 1-night overnight stay).

^f^ Patients receiving urgent treatment either arrived to hospital via ambulance or were admitted through the emergency department.

^g^ Surgery by cancer type did not differ in the 2 cohorts for breast, central nervous system tumors, endocrine, esophagus, genitourinary (not including prostate), prostate, gynecological, head and neck, hepatobiliary, lung, gastric sarcoma, and skin. However, it did increase for colorectal (from 17.2% of cases [87 568 of 509 786] to 21.4% [7254 of 33 965]; standardized difference 0.11) and decrease for melanoma (from 7.7% [39 134 of 509 786] to 5.0% [1694 of 33 965]; standardized difference 0.11).

## Discussion

An immediate 60% decrease in cancer-directed surgery was associated with measures aimed at creating capacity for COVID-19 admissions. This decrease led to major disruptions in cancer care with a large deficit of completed cases in the peri–COVID-19 period compared with the pre–COVID-19 period. Importantly, in a universal publicly funded health care environment, sociodemographic factors were not associated with receipt of surgery in the early peri–COVID-19 period. This suggests equally equitable access to surgical care for patients treated in the early peri–COVID-19 period compared with the pre–COVID-19 period. This study is limited by the lack of timely access to the provincial cancer registry data, which decreased cancer specificity (due to data lag). However, these cancer-directed procedures, whether specifically performed for cancer or not, provide insight to cancer surgical services. Further work is needed to ensure patients experiencing material deprivation are not disadvantaged as cancer diagnostic services increase. The Canadian universal health care context is uniquely positioned to answer cancer outcome questions with an equity lens during the COVID-19 pandemic.
